# Cats and owners interact more with each other after a longer duration of separation

**DOI:** 10.1371/journal.pone.0185599

**Published:** 2017-10-18

**Authors:** Matilda Eriksson, Linda J. Keeling, Therese Rehn

**Affiliations:** Department of Animal Environment and Health, Swedish University of Agricultural Sciences, Uppsala, Sweden; Faculty of Animal Sciences and Food Engineering, University of São Paulo, BRAZIL

## Abstract

Little is known about the cat’s (*Felis silvestris catus*) need for human contact, although it is generally believed that cats are more independent pets than e.g. dogs. In this study, we investigated the effect of time left alone at home on cat behaviour (e.g. social and distress-related) before, during and after separation from their owner. Fourteen privately owned cats (single-housed) were each subjected to two treatments: the cat was left alone in their home environment for 30 min (T_0.5_) and for 4 h (T_4_). There were no differences between treatments in the behaviour of the cat (or owner) before owner departure, nor during the first 5 min of separation. During separation, cats were lying down resting proportionally less (T = 22.5, *P* = 0.02) in T_0.5_ (0.27±0.1 (mean±SE)) compared to in T_4_ (0.58±0.08), probably due to a similar duration of higher activity early in the separation phase in both treatments. Comparisons of the time interval (min 20–25) in both treatments indicated no differences across treatments, which supports such an explanation. Towards the end of the separation phase (the last two 5-min intervals of separation in both treatments), no differences were observed in the cats’ behaviour, indicating that cats were unaffected by separation length. At reunion however, cats purred more (T = 10.5, *P* = 0.03) and stretched their body more (T = 17, *P* = 0.04) after a longer duration of separation (T4:0.05±0.02; 0.03±0.01; T_0.5_: 0.01±0.007; 0.008±0.003). Also, owners initiated more verbal contact (T = 33.5, *P* = 0.04) after 4 h (0.18±0.05) compared to after 30 min (0.12±0.03). There was no evidence of any correlations between the level of purring or body stretching by the cat and verbal contact by the owner implying that the behavioural expressions seen in the cats are independent of the owner’s behaviour. Hence, it seemed as cats coped well with being left alone, but they were affected by the time they were left alone, since they expressed differences in behaviour when the owner returned home. The increased level of social contact initiated by the cats after a longer duration of separation indicates a rebound of contact-seeking behaviour, implying that the owner is an important part of the cat’s social environment.

## Introduction

Cats (*Felis silvestris catus*) are the most common pets in Europe [1], yet little research has been conducted on pet cats in their home setting. One of the reported reasons for their popularity is the expectation about their capacity to cope with being left alone for large parts of the day [2]. However, little is known about how cats are affected by being alone at home during the owner’s working hours, for example. As most present-day owners in Western society are working long hours at the same time as cats are increasing their popularity as pets it is important to address the question of whether or not the time being separated from the owner has any effects on the behaviour and welfare of the cat. This is an important area to investigate considering the high level of separation/isolation related behaviour problems observed among dogs [3, 4, 5], and considering that such responses to owner departure have been seen also in cats [6].

There is a general consensus that the domestic cat is not as solitary as their wild ancestors [7, 8] although there is a large individual variation in how sociable cats are towards humans. These differences are probably due to both genetic influences [9] as well as the level of human handling during the sensitive period [10]. Furthermore, studies suggest that indoor cats initiate more contact with their owners compared to outdoor cats when they are at home [11], which is suggested to be a consequence of a greater need for indoor cats to find different sources of stimulation in the usually quite predictable home environment. Also, as cats normally rest for 16–18 h per day [12], indoor cats may adapt their time awake to activities in the home. Previously, commonly highlighted welfare concerns have been related to cats’ sociality with other cats, such as enforced cohabitation with unfamiliar and unrelated cats or enforced proximity to neighboring cats [13, 14], while their sociality with humans (or the lack of it) is less investigated.

There is conflicting evidence related to cats’ attachment to their owners when cats have been tested using the Ainsworth’s strange situation procedure (ASSP) [15]. Edwards *et al*. [16] found that cats kept solely indoors spent more time in locomotor activities, exploring their surroundings and playing more when they were accompanied by their owner, indicating features of the secure attachment style. They also found that cats vocalised more when they were left alone in the room, which they suggest indicates separation distress. In a counter-balanced version of the ASSP, however, Potter and Mills [17] did not find any evidence of a secure attachment style among cats with outdoor access. Nonetheless, these cats did discriminate between the owner and a stranger, as cats vocalised more when the owner left the room compared to when the stranger left. Besides the difference in experimental design between the two studies, another obvious potential reason for the inconsistent findings could be that there is a difference between how indoor and outdoor cats bond to their owners. Importantly, even if cats generally are not securely attached to their owner, other styles of attachments (ambivalent and avoidant) deserve to be further investigated in order to increase our knowledge about the cat-human relationship [18]. That cats are important social partners for many owners and that humans seem also to be important for many pet cats was explored by Wedl *et al*. [19], who found temporal patterns in interactions between cats and their owners. They interpret their finding, that social interaction patterns varied according to a few major factors thought to influence the relationship quality (e.g. owner and cat personality), as showing that the relationship between the cat and the owner was mutually valuable and beneficial for both partners. It also includes constant ‘negotiations’ of interests [20], which are commonly seen among group-living animals [21]. One indicator of the importance of people to pet cats was found in a longitudinal study of separation anxiety [6]. The study showed that some cats developed separation related behaviour, usually manifested as inappropriate urination and defecation, excessive vocalization, destructiveness and over-grooming, only observed in the absence of the owner. However, the majority of cats do not show abnormal behaviour when separated from their owner, but there is no research investing how these cats respond to being left alone at home.

The effect of time left alone at home on dog behaviour was investigated by Rehn and Keeling [22], using dogs who did not suffer from separation anxiety. They found that while the dog’s behaviour during separation did not change according to the duration of separation, dogs greeted their owner more intensely after longer periods of separation. This indicated that dogs were affected by the time being left alone at home, but it was not expressed until the owner returned. As dogs, many cats are left alone at home for parts of the day and both are carnivore species with long daytime resting periods. Moreover, since there is no evidence to suggest that cats have a poorer concept of time than dogs, in the current study we hypothesise a similar finding, i.e. that there will be differences in how cats respond to the return of the owner (their greeting behaviour) depending on how long he/she has been away, but not necessarily differences in behaviour during the actual separation.

Most animals adapt their greeting behaviour to the situation, which may include the time since the last greeting. For example, greeting sessions are important for confirmation and strengthening of the social bond [23, 24, 25]. Contrary to many other carnivores, cats do not have ritualised submissive signals, which are often seen in dogs greeting their owners [22, 26]. This is probably due to cats originally being more solitary without the need for ‘polite’ appeasement gestures in adulthood. Recognised greeting behaviour in kittens towards the mother are the tail-up posture, followed by head rubbing [27, 28, 29], but also allogrooming and allorubbing between adult cats [7, 30, 31]. Vocalisations, such as the meow and the purr, have been suggested to occur during greeting [32] and contact-maintenance [33]. Hence, these behaviours were of particular interest in the current study.

## Materials and methods

### Subjects

Fourteen privately owned cats (9 females (of which one was intact) and 5 males (all neutered)) and their owners (10 females and 4 males) participated in the study. The age of the cats ranged from 0.6–15.0 (mean±SE; 6.2±1.1) years old. Participants were recruited through advertisements online, on community noticeboards in Uppsala and at the campuses of the Swedish University of Agricultural Sciences and Uppsala University. Except from being healthy and without known behavioural problems, inclusion criteria were that the cats were older than 6 months of age, lived most of their lives indoors and, if they did go out, they were not able to roam outside unsupervised. Owners were asked to sign an informed consent before entering the study and participation was voluntary. All cats had water at libitum, but feeding routines differed between households. Out of the 14 cats, 12 had free access to dry feed. Owners who gave their cats raw/canned feed also reported that this was usually served in the mornings and evenings. All but two owners (of which one owned a cat that did not have free access to feed) stated that it was not usually done in relation to coming home from work. Three digital cameras were used to record the cat at home (two SONY Handycam HDR-CX130 and one CANON LEGRIA HF R 68). One of the cameras covered the entrance area, while the locations of the other two cameras were chosen depending on where the owner believed the cat spent most of the day.

### Data collection and treatments

**Fig 1:**
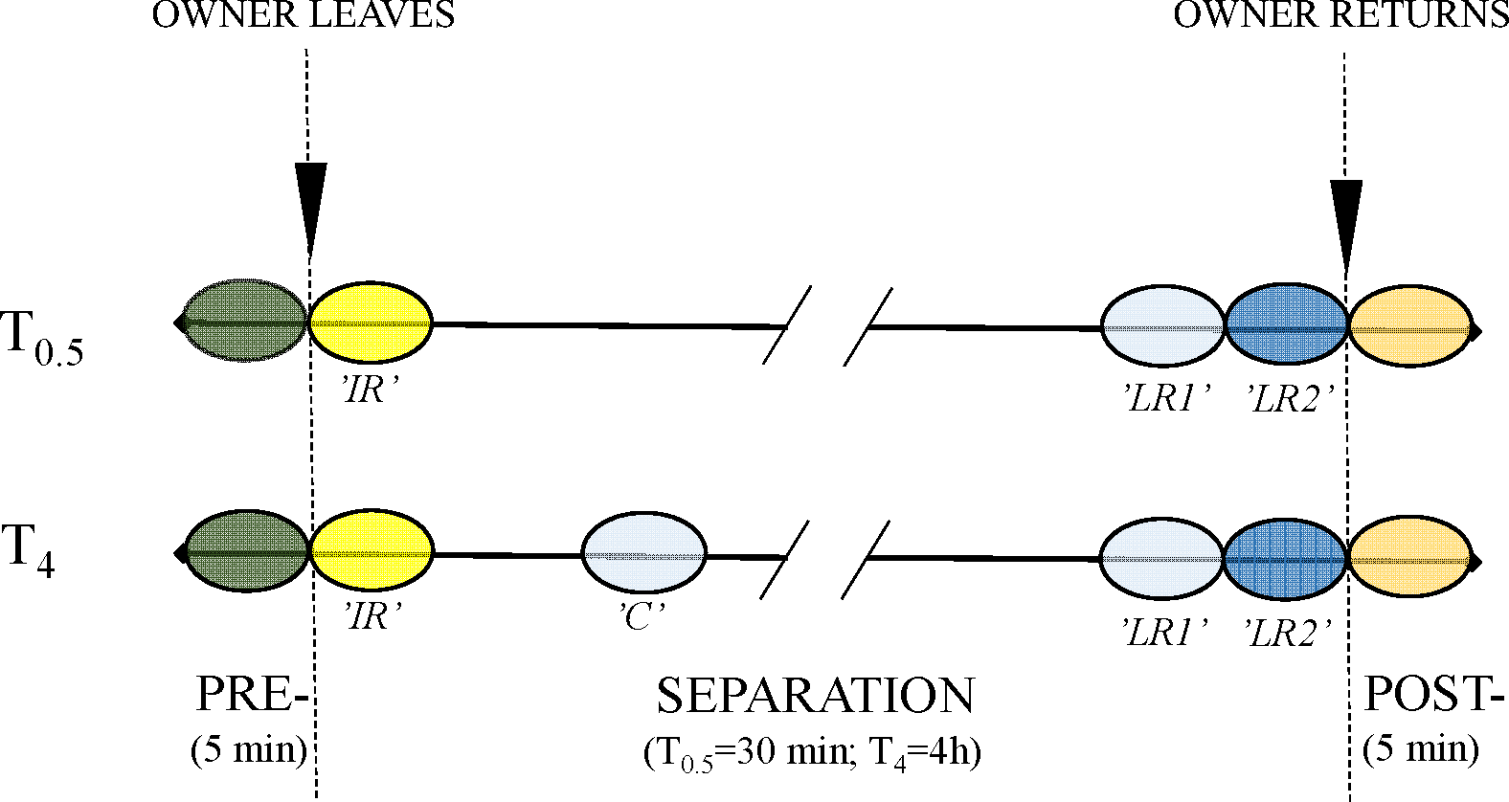
Treatment overview. The behaviour of 14 cats was recorded during two different treatments (T_0.5_: the cat was left alone at home for 0.5 h; T_4_: the cat was left alone at home for 4 h). Data collection started 5 min prior to owner departure (PRE-: pre-separation) and continued until 5 min after the owner returned (POST-: post-separation). Summarised data from different intervals were compared between treatments (circled areas). In addition to the 5-min pre- and post-separation intervals, comparisons were based on one 5-min interval in the beginning (‘IR’: initial response) and two 5-min intervals towards the end of the separation phase (‘LR1’: late response 1; and ‘LR2’: late response 2). Moreover, a 5-min interval occurring at min 20–25 in T_4_ (‘C’: control) was compared to the equivalent time from separation from the owner in treatment in T_0.5_ (‘LR1’). Time intervals with the same colours were compared between treatments.

All data were collected in the cat’s home environment. The behaviour of the cat was recorded on two consecutive days during the same time of the day, either in the morning (sometime between 07:00–12:00) or in the afternoon (12:00–18:00), depending on the owner’s availability. A different separation time was applied on each occasion: the cat was left alone for 30 min (T_0.5_) or for 4 h (T_4_). All cats participated in both treatments and treatment order was equally balanced between the cats. Before data collection started the owner had to be at home for at least 30 min. Data collection started 5 min before the owner left the home (pre-separation) and continued until 5 min after reunion with the owner (post-separation) (Fig 1). Owners were asked to behave as they would normally do towards the cat when leaving and returning home.

Observations of the behaviour of the owner and the cat were made according to the ethogram (S1 Table) from the video-recorded material by a single trained observer using the Interact software (version 2.4 [34]). Behaviour of long duration (e.g. lying, sitting) was recorded instantaneously every 5 s, while behaviour of short duration (e.g. clawing, body shaking) was recorded using one-zero sampling every 5 s. Lip licking, which is easy to count, was recorded using continuous sampling (frequency). If the cat was out of range of the camera, vocalisations were still recorded. Interactions between cat and owner were also recorded, as well as who initiated the (verbal or physical) contact (see S1 Table for details).

### Analyses

In order to investigate the effect of treatment, the recorded period was divided into shorter intervals (Fig 1). Behaviour during the 5-min intervals before the owner left the home (pre-separation) and after the owner returned (post-separation) were summarised and compared between treatments. In the separation phase, data from the 5-min interval immediately following the owner’s departure (initial response, ‘IR’) were analysed and compared across treatments. To investigate the effect of time left alone on cat behaviour, the last two 5-min intervals just before the owner returned were compared (late response 1 (‘LR1’), i.e. the completely undisturbed interval occurring 5–10 min before the owner returned and late response 2 (‘LR2’), i.e. the interval occurring 0–5 min before the owner returned which could be influenced by the owner approaching the home). During the separation phase, behaviour was scored for 10 min in total in each treatment, divided into ten intervals of 1 min each, evenly distributed across the remaining time slot. In addition, an interval (‘C’) at min 20–25 in T_4_ was compared to ‘LR1’ in T_0.5_ (i.e. the equivalent time into the separation phase in both treatments), to control for possible general differences in activity at the beginning of separation. Comparisons of intervals made across treatments are indicated by coloured circled areas in Fig 1.

Behavioural data are presented as the mean proportion of sample points per interval and cat. Statistical analyses were carried out using SAS® computer package (version 9.4). Wilcoxon signed rank tests and Spearman rank correlations were performed as the data were not normally distributed.

### Ethics statement

Since this was an observational study of already existing situations in the life of the cat, no ethical permit for the cats was needed according to Swedish animal welfare legislation (SJVFS 2015:38) nor for the owners participating (SFS 2003:460). However, owners were asked to sign an informed consent before volunteering to participate in the study. This agreement clearly stated that they were allowed to withdraw from the study at any point without any further explanation.

## Results

Out of the total 17.5 h of observed video recorded material, cats were out of camera view for 29.2% of the time. There were no differences in time out of view between the intervals or between the treatments.

### Pre-separation phase

As expected, there were no differences between treatments in the behaviour of the cats or the owners before the owners left the home.

### Separation phase

There were no treatment differences in the cats’ behaviour during the first 5-min interval (‘IR’) after the owner had left the home.

Based on the analyses of the 1-min intervals evenly distributed across the separation phase (giving a total of 10 min of observations), cats were lying down resting more (N = 14, T = 22.5, *P* = 0.02) in T_4_ (0.58±0.08 (mean proportion of sample points ± SE)) compared to during T_0.5_ (0.27±0.1).

There were no differences between treatments in the last two 5-min intervals just before the owner returned (‘LR1’ and ‘LR2’), nor between the intervals occurring at min 20–25 after owner departure (‘C’ in T4 vs. ‘LR1’ in T0.5).

### Post-separation phase

**Fig 2:**
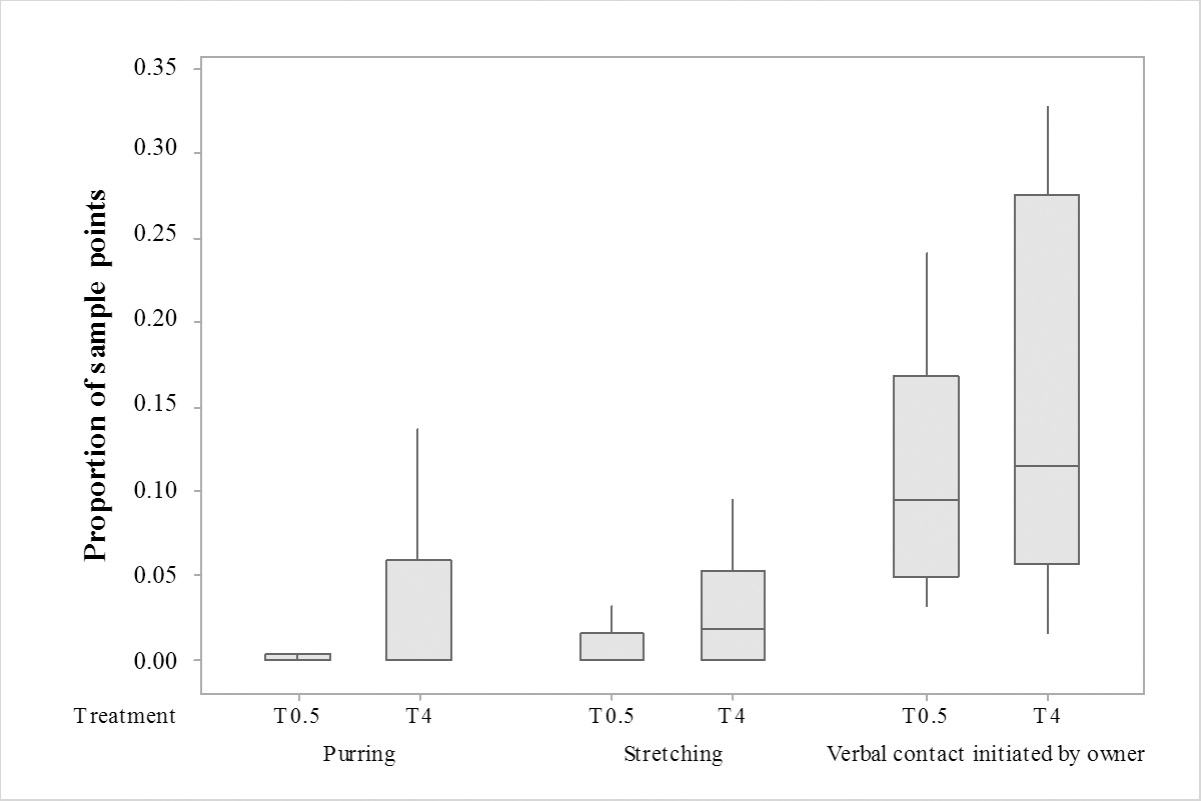
Behaviour at reunion. Boxplot (medians with 95% confidence intervals) showing that cats (N = 14) purred and stretched more at reunion (post-separation phase) and that owners initiated more verbal contact after a longer time of separation. T0.5 = 30 min separation treatment; T4 = 4 h separation treatment.

At reunion with the owner, cats purred more (N = 14, T = 10.5, *P* = 0.03) and showed more body stretching (N = 14, T = 17, *P* = 0.04) after being separated for 4 h compared to when left alone for only 30 min (Fig 2). A positive correlation between purring and body stretching was found in T_0.5_ at reunion (N = 14, Spearman’s rho = 0.69; *P* = 0.007) but not in T_4_. Owners initiated more verbal contact with their cats (N = 14, T = 33.5, *P* = 0.04) after having been away for a longer duration of time. Neither purring nor body stretching correlated with verbal contact initiated by the owner in any of the treatments.

Considering the differences in feeding routines, the two cats who were not fed *ad lib* and one cat who was used to being fed when the owner came back home from work, were excluded from the analyses of the post-separation phase. The new analyses showed similar differences as found for all cats (N = 14, which are reported above) regarding purring (N = 11; T = 10.5; *P* = 0.03) and body stretching (N = 11; T = 12, *P* = 0.05). However, the difference considering the owner initiating more verbal contact with the cat after a longer time of separation disappeared (N = 11; T = 15; *P* = 0.206).

## Discussion

Because the cats’ behaviour during separation did not differ between treatments, there were no signs of cats being affected by the time left alone, but at reunion with the owner cats purred more and showed more body stretching after a longer duration of separation. Also, owners initiated more verbal contact with their cats after a longer separation period. This may indicate an increased motivation to re-establish the relationship after a longer duration of separation. The lack of correlation between the responses of the owner and the responses of the cat imply that these were independent of one another. It supports the hypothesis that cats were more interactive with their owners after a longer duration of separation, but also revealed that owners increased their own contact-seeking behaviour towards the cat by talking more.

While domestic cats are more vocal towards humans in general compared to feral cats and during cat-cat interactions [35, 36], literature suggests that meowing is related exclusively to communication, whereas purring is a general sign of contentment or a care soliciting behaviour [37]. Although purring can occur in many different contexts, even in the clinic when the cat is in pain or is distressed [38]. Hence, it has been suggested that purring may be functioning as a ‘manipulative’ contact- and care-soliciting signal, perhaps derived from when the kitten solicits care from the mother [33], e.g. when separated from [27] or reunited with her [39]. It is unlikely that cats in the current study experienced pain or distress when reunited with the owner in this study, thus it is most likely that they are more solicitous of attention after longer durations of separation. The motivational background for the increased purring seems to be related to the social aspect of the owner coming back, not food, as most cats were fed *ad lib* and the additional analyses, where cats that did not have free access to feed were excluded, showed a similar response pattern. This supports the findings of Vitale Shevre et al. [40] who observed that, although a high individual variation, cats seem to prefer social contact with humans, even over food.

To our knowledge, body stretching in cats has not been scientifically investigated and its possible significance in terms of e.g. greeting situations, has not been addressed previously. Rather, stretching has been assumed to stimulate blood circulation after being stationary for some time and the increase in body stretching after the longer separation duration might be an effect of a longer resting period before the reunion event. That there was a correlation between purring and stretching in T_0.5_ suggests that these behaviours are expressed in synchrony, but this was not the case in T_4._

Despite being more vocal when reunited with their owners after a longer duration of separation, in contrast to dogs [22], cats did not initiate more physical contact. Stroking and rubbing their head, flank and tail towards another individual is a commonly described greeting behaviour in cats with the aim to exchange odours [13]. It has been observed that outdoor cats rub against their owner more often than do indoor cats [41], perhaps related to an increased need to exchange odours and mark their territory after being away from the home. Supported by the results in the current study, one could further speculate that this urge is not elicited when it is the owner who was away from the home and comes back.

A noticeable difference between the results in this study of cat owners and the previous study including dogs and their owner [22], was that the dog owners did not alter their behaviour according to the time separated from their dog while cat owners did. In contrast to dog owners, cat owners increased their level of interaction with the cat after a longer duration of separation. Findings related to fundamental differences between dog and cat people (temperament, personality etc) have been inconsistent and sometimes conflicting (e.g. [42, 43]). In a larger study, Gosling *et al*. [44] found personality differences between self-identified dog and cat persons in all dimensions of the Big Five Inventory. Results showed that cat people scored higher in neuroticism and openness, while dog people scored higher on extraversion, agreeableness, and conscientiousness. That cat people score higher than dog people on the neuroticism scale is supported by Reevy and Delgado [45] who also found that neuroticism among pet owners was positively correlated with a higher level of anxious attachment to pets. Moreover, a study investigating the level of neuroticism in dog owners in relation to dog training showed that the more neurotic the owner was, the more commands (both verbal and gestural) they used during training [46]. One can speculate that the combined experience of slightly more neurotic owners being away from their cats for a longer duration of time might cope with their anxious attachment to their pet, by interacting more with it at reunion, more than would dog owners following a similar period of separation. A more detailed investigation of how owners experienced the reunion with their cat might shed some light on this. Mertens [41] showed that vocal communication by the owner was the most commonly observed behaviour directed towards the cat in the home environment and our study supports that this seems to be considered an important way of communicating with the cat also at reunion after a longer duration of separation. Noteworthy though is that when analyses of owner behaviour were based on owners who fed their cats *ad lib* only, differences between treatments in owner greeting behaviour disappeared. This may indicate that it was the owners who usually gave their cat feed when arriving home who were more interactive.

There is no evidence from this study that cats were aware of the passing of time while the owner was away. Nevertheless, the results suggest that their motivation for social contact changed according to the time that had passed when their owner returned, indicating that cats discriminated between the two time intervals. There is considerable evidence that animals are able to associate to a resource even if they cannot see it [47, 48]. Thus it is possible that cats ‘miss’ their owner, but there is no evidence from the time intervals used in this study that this is experienced negatively.

The current study has a few limitations that we would like to address considering future investigations in the area. Firstly, because of the obvious differences in separation duration, the observer was not blind to the treatment she was coding off. Editing the collected video material into equally long periods would have been a better approach. Secondly, although this study was hypothesis driven in the choice of intervals tested and behaviours included, caution should be taken when interpreting the results due to the large number of behaviours tested. We therefore encourage further work on how cats are affected by being home alone. Finally, in this study cats were only recorded once in each treatment. Also, an interesting aspect in future studies would be to include replicates from each individual cat in order to demonstrate the consistency of behaviour within cats and conditions.

## Conclusion

In summary, this study revealed that cats interact more intensely at reunion with the owner by purring more after a longer duration of separation which may reflect the greater need to re-establish the relationship between cat and owner after the longer separation. Whether stretching is a part of this or merely a consequence of longer inactivity remains to be investigated. Alternatively, cats may be responding to the owners’ vocalisations, but the lack of correlations between these cat and human responses at reunion imply they are independent of one another. This was further supported by results based on *ad lib* fed cats only. The social role of humans in the lives of cats deserves more attention in future studies and, since cats behaved differently towards the owner according to the time being separated, the common notion about cats being able to cope well alone at home should be evaluated further. Of practical interest would be to investigate longer separation times, as these would better reflect the typical situation for most pet cats with working owners.

## Supporting information

S1 Table Ethogram. List of the behaviours recorded before (cat and owner), during (cat) and after separation (cat and owner), their definitions as well as recording method.
(DOCX)
