# Supplementary material for: Cats and owners interact more with each other after a longer duration of separation
Source: PLoS One. 2017 Oct 18;12(10):e0185599. doi: 10.1371/journal.pone.0185599 (PMC5646762; doi:10.1371/journal.pone.0185599)
Supplement: S1 Table — List of the behaviours recorded before (cat and owner), during (cat) and after separation (cat and owner), their definitions as well as recording method. (DOCX) [file pone.0185599.s001.docx]

**S1. Table 1. Ethogram.** List of the behaviours recorded before (cat and owner), during (cat) and after separation (cat and owner), their definitions as well as recording method

| Behaviour | Definition | Recording method |
| --- | --- | --- |
| Location: Hallway | Cat (more than 50% of the body) is located in the hallway | Instantaneous (5s) |
| Location: Window | Cat (more than 50% of the body) is located in a window | Instantaneous (5s) |
| Walking/trotting | Cat is moving in a walking or trotting pace | Instantaneous (5s) |
| Standing | Cat is standing still on its paws | Instantaneous (5s) |
| Sitting | Cat is sitting down | Instantaneous (5s) |
| Lying alert | Cat is lying down, head not in contact with floor/furniture | Instantaneous (5s) |
| Lying resting | Cat is lying down, head in contact with floor/furniture | Instantaneous (5s) |
| Exploring | Cat is sniffing/licking/biting/ manipulating something (not toys) | 1/0-sampling (5s) |
| Attention towards door | Cat is focused on the door, by gazing/staring at the door (<2 sec) | 1/0-sampling (5s) |
| Attention towards owner | Cat is focused on the owner, by gazing/staring at the owner (<2 sec) | 1/0-sampling (5s) |
| Attention outdoors | Cat is focused on the outdoors, by gazing/staring at the outdoors through e.g. a window (<2 sec) | 1/0-sampling (5s) |
| Attention towards unidentified | Cat is focused on an unidentified object, by gazing/staring at it (<2 sec) | 1/0-sampling (5s) |
| Playing with object | Hunt-like postures, galloping, manipulating objects with paws, directed towards toys or other objects | 1/0-sampling (5s) |
| Playing solitary | Hunt-like postures and galloping, not directed towards specific objects/person | 1/0-sampling (5s) |
| Playing with owner | Hunt-like postures, galloping, manipulating objects with paws, directed towards owner, or object manipulated by owner | 1/0-sampling (5s) |
| Grooming | Cat is cleaning its body surface by licking, nibbling, picking, rubbing, scratching etc. | 1/0-sampling (5s) |
| Physical contact with object | Cat is rubbing its face or body against objects | 1/0-sampling (5s) |
| Physical contact with owner | Cat is rubbing its face or body against the owner (cat initiated contact) | 1/0-sampling (5s) |
| Meowing | Cat is performing a high-pitch meow | 1/0-sampling (5s) |
| Purring | Cat is performing a low-pitch purring | 1/0-sampling (5s) |
| Tail-up | Tail is in vertical position | 1/0-sampling (5s) |
| Tail wagging | Cat is wagging its tail from side to side | 1/0-sampling (5s) |
| Verbal contact owner | Owner initiates verbal contact with cat | 1/0-sampling (5s) |
| Physical contact owner | Owner initiates physical contact with cat (e.g. petting, lifting up) | 1/0-sampling (5s) |
| Near door | Cat is within 1 m of the front door | 1/0-sampling (5s) |
| Near owner | Cat is within 1 m of the owner | 1/0-sampling (5s) |
| Arching | Cat is arching its back (note if pilo-erection) | 1/0-sampling (5s) |
| Body stretching | Cat is extending/stretching a part of or the whole body | 1/0-sampling (5s) |
| Yawning | Cat is yawning | 1/0-sampling (5s) |
| Body shaking | Cat shakes a part of or the whole body | 1/0-sampling (5s) |
| Lip licking | Cat is licking its lips | Frequency (5s) |
| Treading | Cat is treading its paws, similar to the way they tread while suckling at a young age | 1/0-sampling (5s) |
| Clawing object | Cat is manipulating an object using its claws | 1/0-sampling (5s) |
